# Modelling and Experimental Verification of Step Response Overshoot Removal in Electrothermally-Actuated MEMS Mirrors

**DOI:** 10.3390/mi8100289

**Published:** 2017-09-25

**Authors:** Mengyuan Li, Qiao Chen, Yabing Liu, Yingtao Ding, Huikai Xie

**Affiliations:** 1 School of Information and Electronics, Beijing Institute of Technology, Beijing 100081, China; ytd@bit.edu.cn; 2 Department of Electrical and Computer Engineering, University of Florida, Gainesville, FL 32611, USA; 3 WiO Technology Co., Ltd., Wuxi 214035, China; wio@wiotek.com (Q.C.); ybliu@wiotek.com (Y.L.)

**Keywords:** micro-electro-mechanical system (MEMS) mirror, bimorph, electro-thermal actuator, resonance frequency, thermal modelling, overshoot, ringing

## Abstract

Micro-electro-mechanical system (MEMS) mirrors are widely used for optical modulation, attenuation, steering, switching and tracking. In most cases, MEMS mirrors are packaged in air, resulting in overshoot and ringing upon actuation. In this paper, an electrothermal bimorph MEMS mirror that does not generate overshoot in step response, even operating in air, is reported. This is achieved by properly designing the thermal response time and the mechanical resonance without using any open-loop or closed-loop control. Electrothermal and thermomechanical lumped-element models are established. According to the analysis, when setting the product of the thermal response time and the fundamental resonance frequency to be greater than Q/2π, the mechanical overshoot and oscillation caused by a step signal can be eliminated effectively. This method is verified experimentally with fabricated electrothermal bimorph MEMS mirrors.

## 1. Introduction

Micro-electro-mechanical system (MEMS) mirrors were reported to be in use as early as 1980 as an optical scanner [1]. Since then, MEMS mirrors have been used in a wide range of applications, such as optical switches or optical attenuators in telecommunications [2,3], object tracking [4], projection displays [5], and 3D sensing [6]. Different applications may have different requirements for MEMS mirrors, but stable switching or scanning is always needed. For example, in optical switching, MEMS mirrors are the optical engine for high-precision optical beam positioning, which requires fast and stable switching with minimal cross talk between channels [7]. In the application of object tracking, a MEMS mirror is used to steer a laser beam to a target, which requires the laser steering to be accurate, fast and smooth [4]. Mechanically, a typical MEMS mirror can be simply modelled as a spring-mass-damper system, where the mirror plate is the mass. Most MEMS mirrors operate in air or vacuums, which is typically an under-damped condition that will cause undesired oscillation and overshoot when applying a step input. The under-damped oscillation and overshoot will increase the settling time and may also seriously affect the performance of the whole optical system, such as introducing cross talk or missing the target [8,9]. 

Usually, to suppress or remove the under-damped oscillation, a control strategy such as open-loop control or closed-loop control may be employed [10]. Closed-loop control requires position sensing, which increases system complexity and cost [11]. Shaping input signals is an open-loop control method, where a pre-shaped input signal that corresponds to the reverse of the oscillation with proper time delay is constructed and then applied to control the system [12,13,14]. For example, Daqaq et al. employed an input shaping scheme to realize a desired scanning beam locus with an electromagnetic MEMS mirror [14], where the input signal was calculated based on mirror dynamic characteristics, and using the Laplace transform. Shi et al. reported a method to control a thermally-actuated MEMS mirror based on a high-order dynamic model, and the experiment shows the residual oscillation is greatly eliminated [15]. However, the accuracy of the model largely affects the performance of the open-loop control method, and it may not work well when it is a relatively complex system, such as a high-order system, or a time-varying system.

In this paper, we report a solution that can eliminate under-damped oscillation of electrothermal bimorph MEMS mirrors without using either the open-loop or closed-loop control strategy. This solution utilizes the low pass nature of thermal response to suppress the mechanical oscillation. This paper is organized as follows. In Section 2, the electrothermal bimorph actuation principle and the electrothermal bimorph based MEMS mirror design are introduced first, and then the static and dynamic characteristics of a fabricated electrothermal bimorph MEMS mirror with step response overshoot is presented. In Section 3, a thermomechanical model of the bimorph MEMS mirror is developed, from which the new method of suppressing the step response overshoot and oscillation is established. Section 4 presents the design and testing results of the improved electrothermal bimorph MEMS mirror based on the new method, which experimentally validates the effectiveness of the overshoot suppression. 

## 2. Two-Axis Electrothermal MEMS Mirror

**Figure 1:**
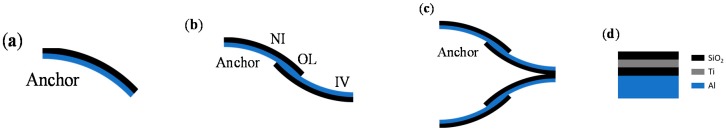
Electrothermal bimorph actuator design. (**a**) Simple bimorph beam; (**b**) inverted-series-connected (ISC) bimorph design; (**c**) Folded double ISC bimorph design; (**d**) A Ti heater embedded in the bimorph.

The two-axis electrothermal MEMS mirror used in this study is based on an inverted-series-connected (ISC) thermal bimorph actuator structure [16]. A thermal bimorph refers to a beam consisting of two layers of materials with different thermal expansion coefficients (TEC). When the temperature of the bimorph changes due to the Joule heating generated by a heater embedded in the bimorph, the bimorph bends because of the TEC difference. However, the tip of a single bimorph has both tangential tip-tilt and lateral shift upon actuation, as shown in Figure 1a. Thus, a unique ISC bimorph actuator design has been developed [17], as shown in Figure 1b, where each ISC bimorph consists of an inverted (IV) segment, a non-inverted (NI) segment, and an overlap (OL) segment, resulting in an “S” shape. Two such bimorphs are connected in a folded fashion, eliminating both tip-tilt and lateral shift at the end of the folded beam, as shown in Figure 1c. The two materials in the bimorph are typically aluminum (Al) and silicon dioxide (SiO_2_) because of their large TEC difference. A titanium (Ti) layer is embedded in the SiO_2_ along the ISC bimorph actuators to form a resistor, as shown in Figure 1d. When voltage is applied to the Ti resistor, Joule heat is generated, which changes the bimorph temperature. 

**Figure 2:**
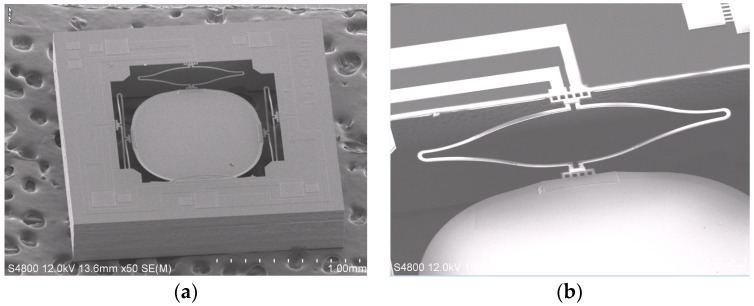
Two-axis ISC MEMS mirror. (**a**) Scanning electron microgram (SEM) of a fabricated device (2 mm × 2 mm); (**b**) SEM of an ISC actuator.

A fabricated two-axis ISC MEMS mirror is shown in Figure 2a, which consists of four ISC bimorph actuators and a mirror plate. The mirror plate is supported by the four ISC actuators on the four sides symmetrically. With four ISC actuators controlling the four sides of the mirror plate, the mirror plate can move vertically or generate angular scan in two axes. The device is fabricated using a hybrid bulk- and surface-micromachining process and SOI wafers are selected to ensure the flatness of the mirror plate. The process includes Ti heater lift-off, SiO_2_ plasma-enhanced chemical vapor deposition (PECVD), Al sputter deposition, SiO_2_ reactive ion etch (RIE), and silicon deep reactive-ion etching (DRIE), as described in [18]. A fabricated device is shown in Figure 2a, where the chip size is 2 mm × 2 mm, and the diameter and thickness of the mirror plate are 1 mm and 25 μm, respectively. A zoom-in view of an ISC actuator is shown in Figure 2b, which shows a clear “S” shape. There is a Ti resistor running along the entire bimorph loop. The mirror plate is 150 μm below the substrate surface.

## 3. Step Response Modelling of the Electrothermal Bimorph MEMS Mirror

When a current is injected into the integrated Ti resistor, the bimorph actuator bends due to the different TEC of Al and SiO_2_. The transfer function of the dynamic response of the MEMS mirror can be expressed as

(1) $$H\left(s\right)=H_{T}\left(s\right)\cdot H_{M}\left(s\right)$$

where *H_T_*(*s*) and *H_M_*(*s*) are, respectively, the transfer functions of the electrothermal response, and mechanical response of the bimorph actuator. 

Firstly, these two physical processes will be modelled separately. According to the study reported in [19], the temperature is quite uniform on bimorph actuators since the resistive heater is uniformly distributed along the bimorph, and the thermal isolations on both ends of the bimorph are good. Thus, the electrothermal response of the bimorph actuator can be approximately modelled as a first-order system. The corresponding heat transfer equation can be simplified as:

(2) $$C_{T}\frac{d\Delta T}{dt}+\frac{\Delta T}{R_{T}}=P$$

where ΔT is the average temperature change on the bimorph, $C_{T}$ is the heat capacitance of the bimorph actuator, $R_{T}$ is the equivalent thermal resistance from the bimorph to the substrate and the ambient, and P is the input electrical power. From Equation (2), the transfer function of the thermal response of the system can be derived as:

(3) $$H_{T}\left(s\right)=\frac{R_{T}}{sR_{T}C_{T}+1}$$

**Figure 3:**
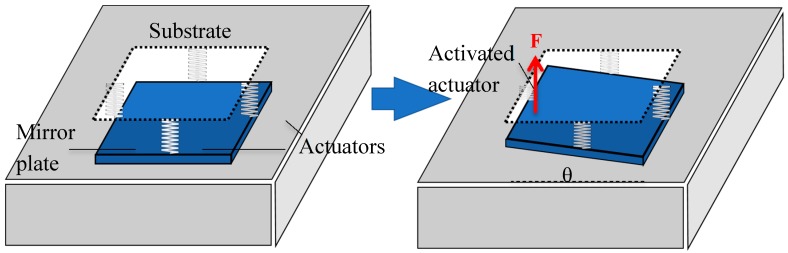
The mass-spring model of the MEMS mirror. Only the bimorph actuator on the left is activated.

Let us consider the case when only one actuator is activated. In this case, the opposing actuator will be stationary while the two neighboring actuators will be displaced as much as half of that of the activated actuator, as illustrated in Figure 3. 

Thus, the mirror can be modelled as a second-order mass-spring-damper system. The corresponding equation of motion is given by:

(4) $$I\frac{d^{2}\theta }{dt^{2}}+D\frac{d\theta }{dt}+k_{\theta }\theta =FL$$

where *I* is the moment of inertia of the square mirror plate, θ is the rotation angle of the mirror plate, *D* is the air damping coefficient, $k_{\theta }$ is the equivalent torsional stiffness of all the bimorph actuators combined, *L* is length of the mirror plate, and *F* is the force generated by the activated actuator. Thus, the mechanical force-to-angle transfer function is readily obtained from Equation (4):

(5) $$H_{M}\left(s\right)=\frac{L/I}{S^{2}+\left(D/I\right)S+k_{\theta }/I}$$

Plugging Equations (3) and (5) into Equation (1) yields

(6) $$H\left(s\right)=H_{T}\left(s\right)\cdot H_{M}\left(s\right)=\frac{R_{T}}{R_{T}C_{T}S+1}\cdot \frac{L/I}{S^{2}+\left(D/I\right)S+k_{\theta }/I}$$

(7) $$\mathrm{or}\;H\left(s\right)=\frac{1}{\tau S+1}\cdot \frac{\omega_{n}{}^{2}}{S^{2}+2\omega_{n}\zeta S+\omega_{n}{}^{2}}=\frac{\frac{1}{\tau }\cdot \omega_{n}{}^{2}}{\left(S^{2}+2\omega_{n}\zeta S+\omega_{n}{}^{2}\right)\left(S+\frac{1}{\tau }\right)}$$

where $\tau =R_{T}C_{T}$, $\omega_{n}=\sqrt{k_{\theta }/I}$, and $\zeta =\frac{D}{2\sqrt{k_{\theta }I}}$ respectively represent the thermal time constant, natural resonant frequency of rotation, and damping ratio of the bimorph-mirror plate system.

For an under-damped system, the normalized step response in time domain can be obtained from Equation (7), i.e.,

(8) $$\tilde{\theta }\left(t\right)=1-\frac{e^{-\beta \zeta \omega_{n}t}}{\zeta^{2}\beta \left(\beta -2\right)+1}-\frac{\zeta \beta e^{-\zeta \omega_{n}t}}{\sqrt{1-\zeta^{2}}\sqrt{\zeta^{2}\beta \left(\beta -2\right)+1}}\sin\left(\omega_{d}t+\alpha \right)$$

where $\beta =\frac{1}{\tau \omega_{n}\zeta }$, $\omega_{d}=\omega_{n}\sqrt{1-\zeta^{2}}$

(9) $$\alpha =\arctan\frac{\zeta \left(\beta -2\right)\sqrt{1-\zeta^{2}}}{\zeta^{2}\beta \left(\beta -2\right)+1}$$

For an under-damping system, it is more convenient to use quality factor $Q=\frac{1}{2\zeta }$ to represent damping; also $\omega_{n}=2\pi f_{0}$, where $f_{0}$ is the resonance frequency. The dynamical response of the whole system mainly depends on these parameters. As shown in (7), the dynamical response of the whole system includes a first-order low-pass filter sub-system and an under-damped second-order sub-system. Typically, the mirror plate of such a MEMS mirror is about 1 mm in size and surrounded by air, leading to a resonance frequency in the range of 0.3–3 kHz, a thermal time constant in the range of 1–50 ms, and a quality factor, or *Q* factor, of about 50 [17,18,20]. So, ζ is about 0.01.

**Figure 4:**
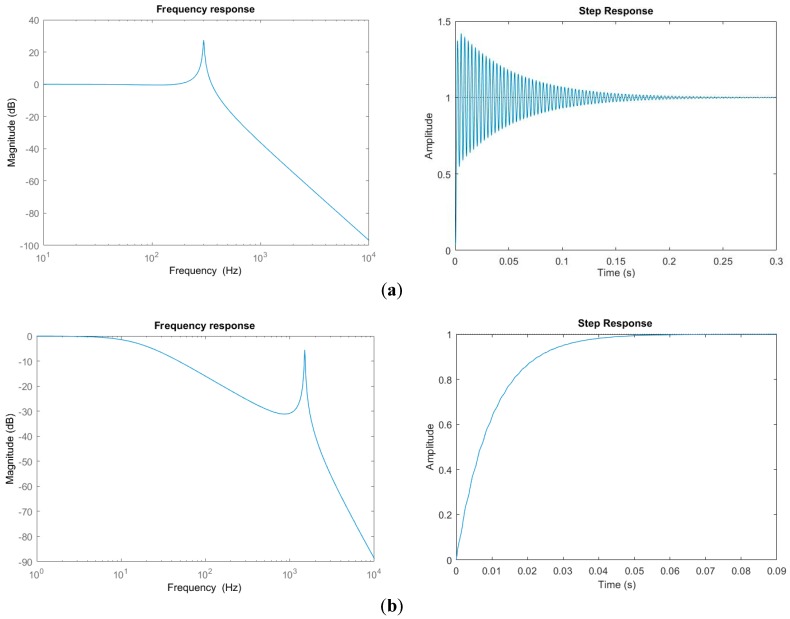
Frequency response and step responses of the whole system with (**a**) *Q* = 50, τ=1 ms and $f_{0}$ = 300 Hz; (**b**) *Q* = 50, τ=10 ms and $f_{0}$ = 1,500 Hz.

Hypothetically, let us take *Q* = 50 and consider different τ and $f_{0}$. For example, take (a) τ=1 ms, and $f_{0}$ = 300 Hz, and (b) τ=10 ms, and $f_{0}$ = 1.5 kHz. Figure 4 shows the frequency response and step response of this system. Not surprisingly, there is overshoot and ringing in the step response as shown in Figure 4a. More interestingly, the step response in Figure 4b exhibits almost zero overshoot and ringing. This indicates that the step response’s overshoot and ringing can be eliminated by properly designing the thermal response and mechanical response of the electrothermal bimorph actuator.

## 4. Elimination of Overshoot and Ringing

According to Equation (3), the cutoff frequency of the thermal response $f_{c,T}$ is given by

(10) $$f_{c,T}=\frac{1}{2\pi \tau }$$

Since the thermal response functions as a low pass filter, the mechanical response will decrease rapidly with increasing frequency above $f_{c,T}$ by 20 dB/decade. Thereby, the peak of the mechanical resonance will be suppressed by a factor of $f_{0}/f_{c,T}$. As the mechanical gain at the resonance is equal to *Q* for an under-damped system, to completely remove the overshoot and ringing, we must have

(11) $$f_{0}/f_{c,T}\geq Q$$

Plugging Equation (10) into (11) yields

(12) $$\tau \cdot f_{0}\geq \frac{Q}{2\pi }$$

**Figure 5:**
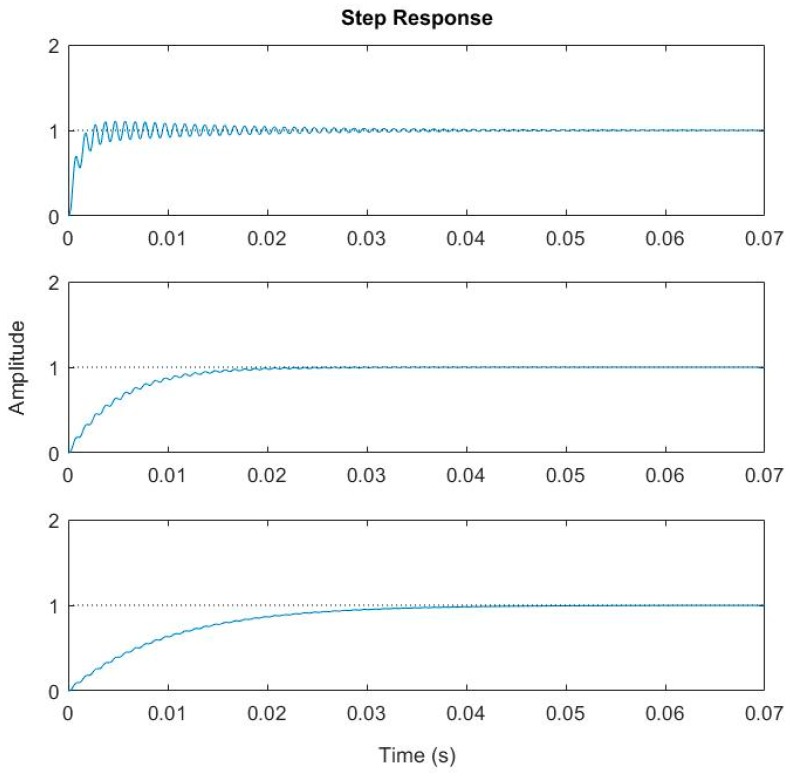
Step response with fixed resonant frequency of 1 kHz and different thermal time constants (τ=1, 5, 10 ms).

**Figure 6:**
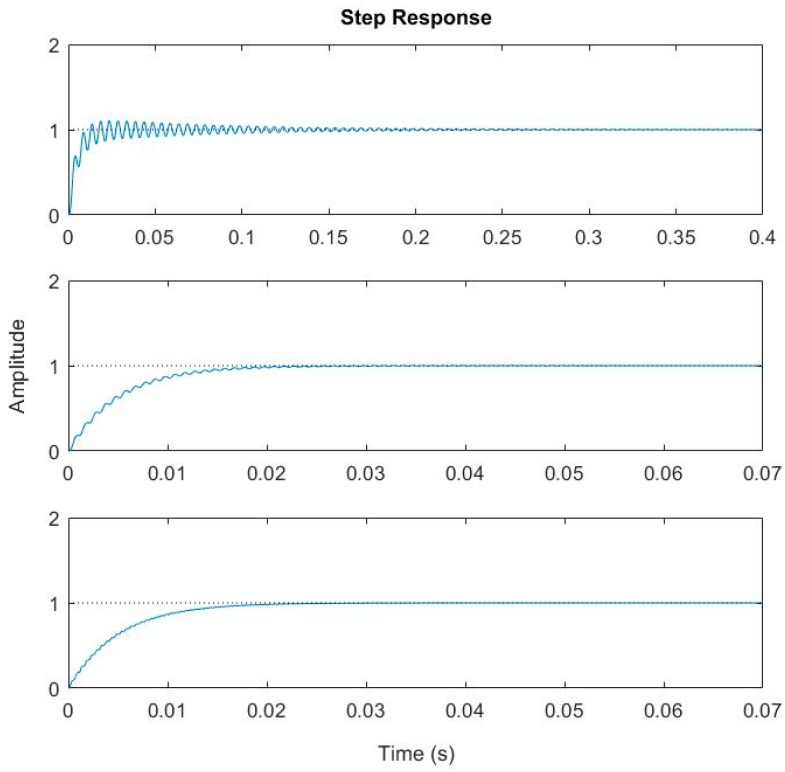
Step response with τ = 5 ms and different resonant frequencies ($f_{0}=$ 0.2, 1.0, 2.0 kHz).

Thus, according to Equation (12), for a given packaging environment (i.e., *Q* is fixed), an electrothermal bimorph actuator with fast thermal response must have high resonant frequency in order to suppress the overshoot and ringing of a step response. Numerical plotting Equation (8) will provide a better understanding. Let us still take *Q* = 50. Figure 5 shows the step response of a system with $f_{0}$ = 1 kHz and τ varying from 1 ms to 10 ms, while Figure 6 shows the step response of a system with τ = 5 ms and $f_{0}$ varying from 200 Hz to 2 kHz. It can be observed that both the overshoot and ringing are effectively removed when τ≥10 ms for $f_{0}$ = 1 kHz or when $f_{0}\;\geq \;$2 kHz for τ=5 ms. In both cases, $\tau \cdot f_{0}>50/\left(2\pi \right)≃8$, which agrees very well with Equation (12). 

## 5. Experimental Verification

**Figure 7:**
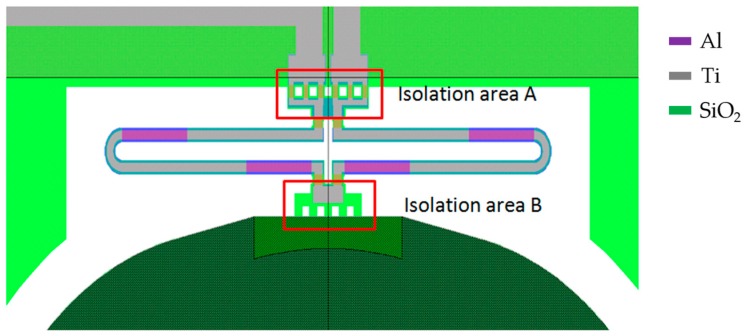
Schematic view of a single bimorph actuator design.

According to the analysis in Section 4, both the thermal response time and the resonant frequency determine the characteristics of the step response of an electrothermal MEMS mirror. Figure 7 shows a schematic diagram of a portion of an electrothermal MEMS mirror, including a complete thermal bimorph actuator, and part of the mirror plate. The bimorph actuator further consists of a folded double ISC bimorph, a thermal isolation A between the bimorph and the substrate, and a thermal isolation B between the bimorph and the mirror plate. 

**Figure 8:**
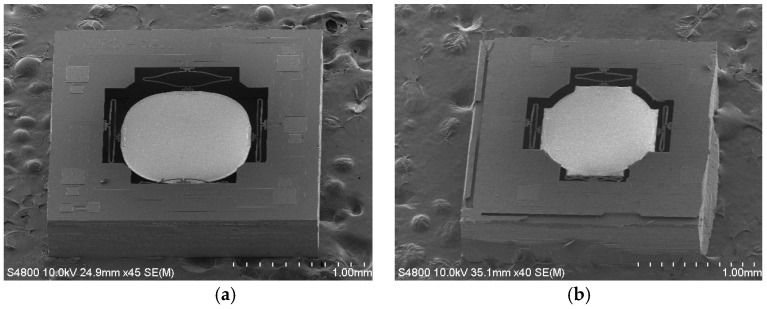
SEMs of two MEMS mirrors. (**a**) Design 1; (**b**) Design 2.

As we described above, the thermal response time and the resonant frequency are given by

(13) $$\tau =R_{T}\;C_{T}\;\mathrm{and}\;f_{0}=\frac{1}{2\pi }\sqrt{k_{\theta }/I}$$

where $R_{T}$ is the inverse of the thermal conduction of the thermal isolation A plus the thermal conduction of the air around the bimorph, $C_{T}$ is the thermal capacitance of the entire bimorph, $k_{\theta }$ is the torsional stiffness of the bimorph, and I is the moment of inertia which is proportional to the mass of the mirror plate. The stiffness of the bimorph $k_{\theta }$ can be changed largely by varying the length of the bimorph. Note that the thermal isolation region B blocks the heat flux from flowing into the mirror plate; it does not affect the thermal response time of the bimorph actuator and its thermal resistance is much greater than $R_{T}$ in Equation (13). Table 1 lists two designs with different bimorph lengths leading to $f_{0}$. Figure 8 shows SEMs of both MEMS mirror designs. 

**Figure 9:**
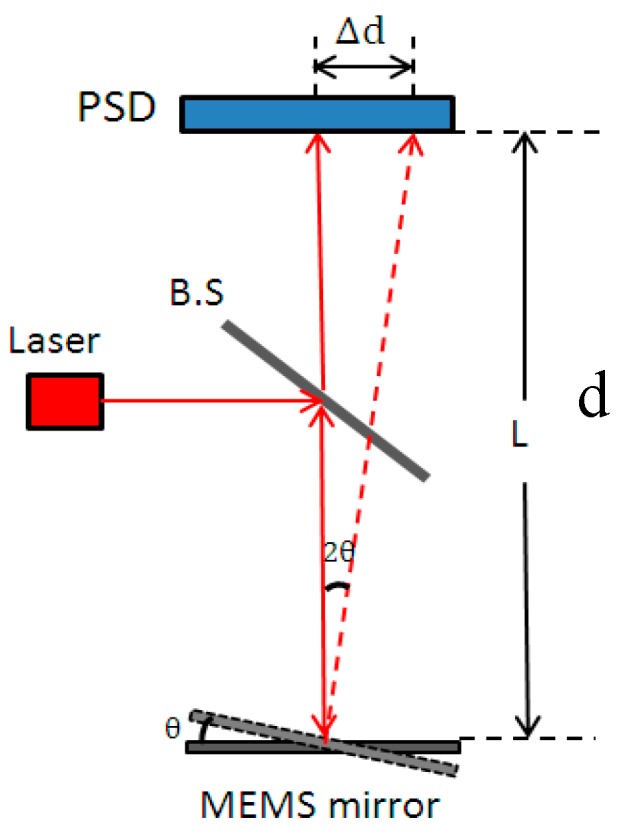
Experimental setup for measuring the mirror tilt angle.

To obtain the step response of the MEMS mirrors, a setup as illustrated in Figure 9 was constructed, where a laser beam was directed to the center of a MEMS mirror through a beam splitter (BS), and then the laser beam was reflected by the MEMS mirror and incident on a position sensitive device (PSD) (OT-302D, On-Trak Photonics, Inc., California, USA). The output signal of the PSD was a direct measure of the lateral shift of the laser spot. The mirror tilt angle is readily calculated as follows:

(14) $$\theta =\frac{1}{2}\arctan\frac{\Delta d}{d}$$

where θ is the mirror tilt angle, d is the distance between the mirror and the PSD, and Δd is the laser spot displacement on the PSD.

**Figure 10:**
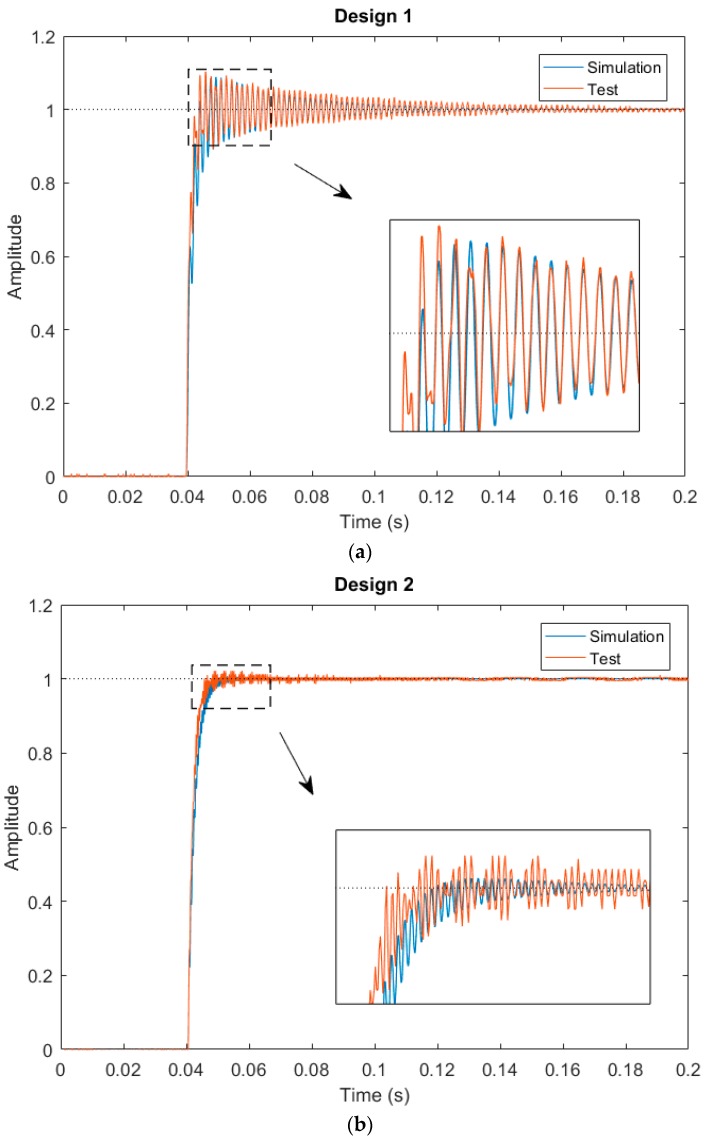
Step responses of the MEMS mirrors. (**a**) Design 1; (**b**) Design 2.

First, the resonant frequencies of the two designs shown in Figure 8 were measured with simple frequency-sweeping using the setup shown in Figure 9, which were 0.592 kHz, and 1.89 kHz, respectively. Then a step voltage signal was applied to one of the actuators of a MEMS mirror, and the PSD output signal was recorded, which was the step response of the MEMS mirror. Figure 10 shows the step responses of the two designs. The experimental rise time $t_{r}$ and $f_{0}$ for the two designs are listed in Table 2. 

For a first order system, the step response is given by $\left(1-e^{-\frac{t}{\tau }}\right)$. Thus, the 10% to 90% rise time can be readily derived as $t_{r}=\left(ln9\right)\tau \approx 2.2\tau$. Using this relation, the τ values are calculated and given in Table 2. Also plotted in Figure 8 are the simulated step responses with *Q* = 50. There is a small difference between the experiment and simulation, which is believed to be due to the fact that the assumed *Q* = 50 may not be accurate. 

As shown in Figure 10 and Table 2, when the product of $\tau \cdot f_{0}$ is increased from 1 to about 4, the overshoot for Design 2 is reduced by a factor of 5. However, the $\tau \cdot f_{0}$ product for Design 2 is still less than 8, when the optimal value is calculated from Equation (12). Thus, just as predicted, a small overshoot remains in Design 2. In order to further reduce the overshoot, $\tau \cdot f_{0}$ must be increased. We may increase either τ or $f_{0}$ or both. Following this study, the bimorph length may be further reduced to increase $f_{0}$. According to Equation (13), other structural parameters, such as the thickness and width of each layer in the isolation and bimorph, the density of the holes on the isolation region, and the thickness and size of the mirror plate, can all be used to tune the product of $\tau \cdot f_{0}$.

## 6. Conclusions

In this work, a solution that can suppress the overshoot and ringing of the step response of an under-damped electrothermal bimorph actuated MEMS mirrors is proposed and experimentally verified. A model based on the dynamical response of the electrothermal MEMS mirror is established. This model shows that the dynamical response of the electrothermal MEMS mirror can be considered as a first-order RC system connected with a second spring-mass system. Simply tuning the product to $\tau \cdot f_{0}$ can reduce, or even completely remove, the overshoot and ringing of the step response of the electrothermal MEMS mirror operating in air. This method provides a powerful venue for optimal use of electrothermal MEMS mirrors. 

## Figures and Tables

**Table 1:** Parameters of two different MEMS mirror designs.

Design Type	Thermal Isolation A Length (μm)	Bimorph Length (μm)	Mirror Diameter (mm)
Design 1	110	845	1
Design 2	85	500	1

**Table 2:** $\tau \cdot f_{0}$ of the two different MEMS mirror designs.

Design Type	Rise Time $t_{r}$ (ms)	Thermal Response Time, *τ* (ms)	Resonant Frequency, $f_{0}$ (kHz)	$\tau \cdot f_{0}$	Overshoot (Test)	Overshoot (Simulation)
Design 1	4.3	1.95	0.592	1.16	10.2%	8.8%
Design 2	4.6	2.09	1.89	3.95	2.2%	0.66%

## References

[1] Petersen K.E. (1980). Silicon torsional scanning mirror. IBM J. Res. Dev..

[2] Tsai J.C., Fan L., Hah D., Wu M.C. A High Fill-Factor, Large Scan-Angle, Two-Axis Analog Micromirror Array Driven by Leverage Mechanism. Proceedings of the IEEE/LEOS International Conference on Optical MEMS and Their Applications.

[3] Fan K.C., Lin W.L., Chiang L.H., Chen S.H., Chung T.T. (2009). A 2 × 2 Mechanical Optical Switch with a Thin MEMS Mirror. J. Lightwave Technol..

[4] Milanovic V., Lo W.K. Fast and high-precision 3D tracking and position measurement with MEMS micromirrors. Proceedings of the 2008 IEEE/LEOS International Conference on Optical MEMs and Nanophotonics.

[5] Van Kessel P.F., Hornbeck L.J., Meier R.E., Douglass M.R. (1998). A MEMS-based projection display. Proc. IEEE.

[6] Ito K., Niclass C., Aoyagi I., Matsubara H., Soga M., Kato S., Maeda M., Kagami M. (2003). System Design and Performance Characterization of a MEMS-Based Laser Scanning Time-of-Flight Sensor Based on a 256 × 64-pixel Single-Photon Imager. IEEE Photonics J..

[7] Lee C.D., Huang L.S., Kim C.J., Wu M.C. (1999). Free-space fiber-optic switches based on MEMS vertical torsion mirrors. J. Lightwave Technol..

[8] Chu P.B., Lee S.S., Park S. (2002). MEMS: The path to large optical crossconnects. IEEE Commun. Mag..

[9] Kim J., Nuzman C.J., Kumar B., Lieuwen D.F. (2003). 1100 × 1100 port MEMS-based optical cross-connect with 4-dB maximum loss. IEEE Photonics Technol. Lett..

[10] Borovic B., Liu A.Q., Popa D.O. (2005). Open-loop versus closed-loop control of MEMS devices: Choices and issues. J. Micromech. Microeng..

[11] Lani S., Bayat D.Z., Despont M. 2D tilting MEMS micro mirror integrating a piezoresistive sensor position feedback. Proceedings of the SPIE OPTO 2015.

[12] Pal S., Xie H. (2010). Pre-Shaped Open Loop Drive of Electrothermal Micromirror by Continuous and Pulse Width Modulated Waveforms. IEEE J. Quantum Electron..

[13] Popa D.O., Kang B.H., Wen J.T., Stephanou H.E., Skidmore G., Geisberger A. Dynamic modeling and input shaping of thermal bimorph MEMS actuators. Proceedings of the 2003 IEEE International Conference on Robotics and Automation (Cat. No. 03CH37422).

[14] Daqaq M.F., Reddy C.K., Nayfeh A.H. (2008). Input-shaping control of nonlinear MEMS. Nonlinear Dyn..

[15] Shi M., Zhang H., Chen Q. The input shaping control of electro-thermal MEMS micromirror. Proceedings of the 2014 IEEE International Conference on Mechatronics and Automation.

[16] Todd S.T., Xie H. (2008). An Electrothermomechanical Lumped Element Model of an Electrothermal Bimorph Actuator. J. Microelectromech. Syst..

[17] Jia K., Pal S., Xie H. (2009). An Electrothermal Tip–Tilt–Piston Micro-mirror Based on Folded Dual S-Shaped Bimorphs. J. Microelectromech. Syst..

[18] Chen Q., Zhang H., Zhang X., Xu D., Xie H. Repeatability Study of 2D MEMS Mirrors Based on S-shaped Al/SiO_2_ bimorphs. Proceedings of the 8th Annual IEEE International Conference on Nano/Micro Engineered and Molecular Systems.

[19] Pal S., Xie H. (2009). A parametric dynamic compact thermal model of an electrothermally actuated micromirror. J. Micromech. Microeng..

[20] Liu L., Pal S., Xie H. (2012). MEMS mirrors based on a curved concentric electrothermal actuator. Sens. Actuators A Phys..

